# Mediterranean diet and its relationship with oral pathology

**DOI:** 10.2340/aos.v85.46815

**Published:** 2026-09-07

**Authors:** Daniela Sánchez Cousiño, Sonia Egido-Moreno, Beatriz González-Navarro, Antonio Francisco López-Sánchez, Javier Fernández-Farhall, Jesús Rodríguez-Molinero, José López-López

**Affiliations:** a Master in Medicine, Surgery, and Oral Implantology, Faculty of Medicine and Health Sciences, University of Barcelona, Barcelona, Spain; b Faculty of Medicine and Health Sciences (Dentistry), University of Barcelona, Barcelona, Spain; c Department of Odontostomatology, Faculty of Medicine and Health Sciences (Dentistry), University of Barcelona, Barcelona, Spain; d Department of Nursery and Stomatology, Faculty of Health Sciences, Rey Juan Carlos University, Madrid, Spain; e High-Performance Research, Development and Innovation Group in Dental Biomaterials of Rey Juan Carlos University, Madrid, Spain; f Lecturer of the Master’s Degree in Periodontology and Osseointegration, Faculty of Health Sciences, Rey Juan Carlos University, Madrid, Spain; g Facultative Director and Head of the Medical Surgical Area of the Dental Hospital of the University of Barcelona (HOUB), L’Hospitalet de Llobregat, Barcelona, Spain; h Department of Odontostomatology, Faculty of Medicine and Health Sciences (Dentistry), University of Barcelona, Barcelona, Spain; i Oral Health and Masticatory System Group, Institut d’Investigació Biomédica de Bellvitge IDIBELL, (Bellvitge Institute of Biomedical Research), L’Hospitalet de Llobregat, Barcelona, Spain

**Keywords:** Mediterranean diet, cardiovascular diseases, dental caries, diabetes mellitus, periodontitis

## Abstract

**Objective:**

To evaluate whether adherence to the Mediterranean Diet (MD) is associated with a lower prevalence of oral and systemic diseases and to investigate the relationship between MD adherence and oral health parameters, including dental caries, periodontal disease, salivary flow, and salivary pH, in a multicenter population of adults.

**Materials and methods:**

We enrolled 232 patients, recorded their medical histories, and assessed their adherence to the MD using the “Mediterranean Diet Adherence Screener” questionnaire. We also randomly performed an intraoral examination on 43 patients from the adherence group and 30 patients from the non-adherence group.

**Results:**

We found a statistically significant difference in the prevalence of systemic conditions, such as cancer (*p* = 0.045) and cardiovascular disease (*p* = 0.02), between patients adhering to the MD and those who did not. Additionally, stimulated pH (*p* = 0.019), periodontal disease stage (*p* = 0.009), decayed, missing, and filled teeth (*p* = 0.048), and stimulated sialometry (*p* = 0.002) were also correlated with MD adherence.

**Conclusions:**

This study found that adherence to the MD acts as a protective factor against both oral and systemic diseases. An associative relationship exists between certain oral and systemic diseases and adherence to the MD.

## Introduction

Traditionally, the Mediterranean diet (MD) is characterized by a high consumption of olive oil, fruits, nuts, vegetables, and grains; a moderate intake of fish and poultry; a low consumption of dairy, red meat, processed meats, and sweets; and wine in moderation, consumed with meals [1]. Therefore, extra virgin olive oil and nuts are probably responsible for most of the observed benefits of the MD [2].

The MD has been shown to promote an antioxidant and anti-inflammatory environment due to the presence of nutrients such as unsaturated fatty acids like omega-3 and antioxidants. Systemic health is closely related to nutrition; it influences craniofacial development and various diseases [2].

Carbohydrates and monounsaturated fatty acids (MUFAs) are among the dietary components that can elevate arterial hypertension (AHT), lipid profiles (low-density lipoprotein [LDL] cholesterol), triglycerides, vascular inflammation, and insulin resistance, all of which are risk factors for cardiovascular disease (CV) [3]. In the trial by Estruch et al. [1], it was observed that adherence to an MD, without caloric restriction but supplemented with extra virgin olive oil or nuts, led to a 30% relative risk reduction in high-risk individuals initially free from CV disease. Additionally, insulin resistance and diabetes are associated with excessive energy intake, particularly from saturated fatty acids and simple sugars, which contribute to increased adiposity [3]. A meta-analysis by Schwingshackl et al. [4] found that higher adherence to the MD is linked to a 19% reduction in the risk of type 2 diabetes. The study emphasizes replacing certain condiments with olive oil, as well as increasing the consumption of vegetables and nuts, while reducing the intake of red or processed meats [5].

Additionally, several studies have shown the importance of diet in chronic kidney diseases (CKDs). Studies indicated that the possible beneficial effects of adherence to the MD include the prevention of albuminuria and reductions in serum levels of urea and creatinine. Plant-derived foods (fruit, vegetables, legumes, and whole grains) are MD components with a high fiber content, low glycemic index, and load; strong evidence supports the protective effects of these foods against potential CKD risk factors [6]. Furthermore, the anti-inflammatory properties of the MD may improve the development and/or progression of various chronic inflammatory diseases and conditions. A suitable dietary intake might be a useful strategy for improving clinical outcomes in the inflammatory diseases such as asthma [7].

We must not forget the antitumor value of an antioxidant diet. Thus, the consumption of fruits and vegetables, with their antioxidant components and micronutrients (carotenoids, vitamin C, vitamin E, selenium, fiber, and polyphenols), can reduce the development of tumors of the digestive epithelium, breast cancer, cancer of the female genital tract, and urinary tract cancer. Oily fish, along with nuts and seeds such as flax and chia, are important sources of omega-3 fatty acids and their precursors. These fats reduce tumor cell growth, lowering the risk of cancers such as liver cancer and colorectal cancer. Whole grapes (including the skin) contain important antioxidant and anticancer compounds, such as resveratrol and quercetin. Finally, and despite what has already been mentioned, whole grains, on the one hand, and olive oil, with its protective compounds such as oleic acid, and antioxidant and anti-inflammatory compounds such as polyphenols (oleuropein and hydroxytyrosol), constitute a limiting factor for tumor development [8–10].

Moreover, oral health is related to nutrition in many ways; a diet rich in fruits, vegetables, and whole grains high in starch and low in free sugars and fats benefits many aspects of oral health, such as the prevention of caries, periodontal diseases and oral cancer [2, 11].

Appropriate and balanced nutrient intake and metabolism provide the substrates for the normal physiological functions of the human body. Nutrition is considered one of the strongest and most adjustable environmental factors that could be used to reduce the burden of disease during an individual’s entire life. Poor nutrition results in an increased risk of several diseases like cancer, diabetes, and heart diseases. This malnutrition could be caused by environmental factors, like food scarcity, as well as disease conditions, like anorexia nervosa, impaired digestion, intestinal malabsorption, or other chronic diseases [12].

In addition, nutritional status affects the teeth before eruption, although diet has a greater local effect after eruption. Vitamin D and A deficiencies and protein-energy malnutrition are associated with enamel hypoplasia and salivary gland atrophy, which increase susceptibility to dental caries [2]. The increased frequency of sugar consumption between meals is associated with a marked increase in dental caries. Similarly, fructose has been found to be acidogenic on plaque pH, although less so than sucrose. Similarly, cow’s milk contains calcium, phosphorus and casein, which inhibit caries by affecting plaque pH. Foods that stimulate salivary flow, including whole-grain foods, peanuts and hard cheeses, protect against caries [2].

Nutrition has also been linked to periodontal disease. In periodontitis, a persistent inflammatory state results from aberrant neutrophil activation and the sustained release of proinflammatory mediators, contributing to disease progression. Neutrophils can also release immune complexes and generate oxidative stress, which are associated with systemic conditions such as CV diseases and diabetes mellitus (DM) [13]. This can be mitigated by the intake of dietary antioxidants, including vitamin C, vitamin E, and carotenoids, which help modulate inflammation. Additionally, obesity influences the host immune and inflammatory system, further increasing the risk of periodontitis [3].

Proteins and simple carbohydrates contribute to the establishment of an acidic microenvironment, which stimulates inflammation. Diet plays a crucial role in the oral dysbiosis typical of periodontal disease by providing nutritional substrates for microorganisms, promoting the creation of a microenvironment conducive to the multiplication and survival of certain periodontal pathogens, while inhibiting the growth of others [14]. Even toothpastes that contain extra virgin olive oil, a key component of the MD, have been found effective in modifying the oral microbiome [15].

These alterations in the oral microbiota can also influence the success of dental treatments. Changes in the microbiota on peri-implant surfaces, for example, may lead to implant failure. The use of probiotics, when combined with mechanical debridement, could help reduce peri-implant pockets due to their mechanism of competitive inhibition at adhesion sites [16].

To assess adherence to the MD, there are different questionnaires such as the ‘Mediterranean Diet Adherence Screener’ (MEDAS), which evaluates adherence to the MD with 14 ‘yes’ or ‘no’ questions [17].

In this sense, conducting a study that encompasses the relationship between adherence to the MD and decreased risk of suffering from certain oral or systemic pathologies could lead to an improvement in the habits of the population of the Mediterranean area. Therefore, the aim of this study was to determine whether greater adherence to the MD implies a lower risk of suffering oral or systemic pathologies.

## Methods

### Study description

This cross-sectional, observational multicenter study included patients who visited the Dental Hospital University of Barcelona (HOUB) and the King Juan Carlos University Clinic of Madrid (KJCUC) from November 2022 to June 2024 for routine visits and who agreed to participate in the study. Ethical approval for this study was obtained from the ethics committee of HOUB (protocol number: 2023-008-1).

In the first part of the study, which examined the population’s adherence to the MD and its relationship with systemic pathologies, the sample size was determined based on the study by Miró et al. [18], which established adherence to the MD at 58.9%. Using the sample size calculation formula, a minimum of 168 patients were required. Our sample size was 232 patients based on proportions with a *p* < 0.05. A complete clinical history was taken from those patients who agreed to participate in the study and signed the informed consent. To assess adherence to the MD, the MEDAS questionnaire [13] was given. Adherence to the MD was stratified according to the score: Yes = 1 point and No = 0 points. Based on their total scores, participants were classified into low adherence (< 8 points) or high adherence (≥ 9 points) groups. We then analyzed the data accordingly and obtained the results.

In the second part of the study, we aimed to evaluate the relationship between oral and systemic pathology and adherence to the MD. The sample size was determined based on the study by Bawadi et al. [19], in which adherence to a good healthy eating index was established at 82.6%. Applying the formula for calculating sample size, a minimum of 10 patients were required. Our sample size was 73 patients based on proportions with a *p* < 0.05. Among all patients who completed the questionnaire to determine adherence to the MD, we randomly selected 43 who were adherent and 30 who were non-adherent and performed intraoral examinations.

### Inclusion and exclusion criteria

The inclusion criteria for this study were as follows: patients over 50 years of age who provided written informed consent to participate in the study.

The exclusion criteria included patients who were under 50 years of age and those who did not give their consent to participate in the study.

### Recorded data and study variables

We recorded:

1. Systemic disease data extracted from the medical history.
2. Data related to the oral cavity:

***Odontogram – Decayed, Missing, and Filled Teeth (DMFT) index***: Analyzing the number of teeth with caries (C), absent (A), and filled (O) [20].

***O’Leary plaque index:*** It indicates the percentage of stained smooth surfaces over the total number of tooth surfaces present and is multiplied by 100. Acceptable: less than 20%; deficient: greater than 20% [21].

***Periodontogram:*** A complete periodontogram was performed, excluding the third molars. The probing depth and the clinical attachment loss were measured with the CP-12 probe. The classification of periodontitis was based on the recommendations established by the “World Workshop on the Classification of Periodontal and Peri-implant Diseases and Conditions” [22].

***Salivary secretion***: (a) *Unstimulated salivary secretion*: saliva was deposited at short intervals in a beaker for 5 minutes. The collected content was then measured in a graduated tube. (b) *Stimulated salivary secretion*: The patient chewed a standardized piece of paraffin wax for 5 minutes. Saliva was collected in the glass at short intervals during the chewing period. The contents were then measured in a graduated tube.

***Salivary pH:*** In each of the vessels with accumulated saliva, the salivary pH was measured by means of FILTERLAB® paper strips, whose color was compared with a chromatic scale that establishes a color for each pH value (1–14). The pH of the stimulated and basal saliva was recorded.

The main variables included in the study were the data extracted from the MEDAS questionnaires, which are related to adherence to the MD. The secondary variables are descriptive of the patient, extracted from their clinical history and intraoral examination.

### Statistical analysis

Data were collected using Microsoft Excel® (Microsoft Corp., Redmond, WA, USA). Categorical variables were described by frequency and percentage; numerical variables were described, according to their distribution, as mean and standard deviation or median and minimum-maximum. Bivariate associations between categorical variables were assessed with the Fisher test for dichotomous variables and the Chi-square test for polychotomous variables. Comparison between numerical and categorical variables was carried out according to the distribution of the numerical variable at each level of the categorical variable. For a normal distribution of the numerical variable, Student’s *t*-tests or analysis of variance (ANOVA) were used, and in cases of a non-parametric distribution, the Mann–Whitney or Kruskal–Wallis tests were used. Bivariate logistic regression (BLR) models were adjusted to assess the association between adherence to the MD and the variables that we have studied. The reported measure of association was the odds ratio (OR) with 95% confidence intervals. A *p-*value < 0.05 was considered statistically significant. All analyses were performed with the program Statistical Package for the Social Sciences (SPSS) Statistics version 26 (IBM Corporation, Armonk, NY, USA).

## Results

This study involved 232 patients: 209 patients from the HOUB in Barcelona and 23 from the KJCUC. Furthermore, we randomly selected 73 patients from the total to perform an intraoral examination: 43 with adherence to the MD and 30 without adherence.

### Systemic pathologies in the study population

The first part of our study included 232 patients who completed the MEDAS questionnaire. The mean ages in the adherence and non-adherence groups were 63.21 ± 9.74 and 65.89 ± 10.67, respectively. In the adherence group, males accounted for 48.4% and females accounted for 51.6%, while in the non-adherence group, males made up 44.4% and females made up 55.6%. Table 1 presents the main demographic characteristics and systemic pathologies of the study population. A higher percentage of patients with systemic diseases was observed in the non-adherence group compared with the adherence group. In the bivariate analysis, adherence to the MD was associated with lower odds of CV disease (OR = 0.382, *p* = 0.002) and cancer (OR = 0.336, *p* = 0.045). After adjustment in the binary logistic regression, only CV disease remained significantly associated with MD adherence (adjusted OR = 0.432, *p* = 0.031).

**Table 1:** Demographic characteristics and systemic pathologies in the sample of 232 patients.

Variables	Adherence *n* (%)	Non-adherence *n* (%)	*p*	OR	BLR (*p*)	OR (BLR)
Gender						
Male	60 (48.4%)	48 (44.4%)				
Female	64 (51.6%)	60 (55.6%)	0.320	1.172	0.297	1.336
Age	63.21 ± 9.74	65.89 ± 10.67	0.047		0.341	0.992
Comorbidities						
CV disease	22 (17.7%)	39 (36.1%)	0.002*	0.382	0.031*	0.432
AHT	36 (29%)	43 (39.8%)	0.096	0.618	0.574	0.638
DM	14 (11.3%)	19 (17.6%)	0.190	0.596	0.663	0.692
Renal disease	1 (0.8%)	3 (2.8%)	0.341	0.285	0.284	0.299
Lung disease	6 (4.8%)	10 (9.3%)	0.204	0.498	0.145	0.324
Cancer	5 (4%)	12 (11.1%)	0.045*	0.336	0.151	0.473
Smoking	7 (16.3%)	5 (16.7%)	0.604	0.972	0.062	0.444

Two hundred and nine patients from the HOUB and 23 from the KJCUC. *P-*value (Chi-square test). OR: odds ratio; BLR: binary logistic regression; CV: cardiovascular; AHT: arterial hypertension; DM: diabetes mellitus; HOUB: Hospital of the University of Barcelona; KJCUC: King Juan Carlos University Clinic of Madrid.

* Significant differences between groups.

### Intraoral examination

Seventy-three patients were randomly selected to undergo an intraoral examination. This multicenter part of the study was conducted between the HOUB, where 50 patients were selected, and the KJCUC, where 23 patients were selected. We present the results of the two populations separately and jointly.

For the 73 patients randomly selected, the mean ages in the adherence group and non-adherence group were 60.58 ± 8.06 years and 63.2 ± 10.79 years, respectively. The male and female distribution was 58.1% and 41.9%, respectively, in the adherence group, and 50% and 50% for the non-adherence group.

Table 2 presents the main demographic characteristics, along with oral and systemic pathologies. In the bivariate analysis, adherence to the MD was associated with lower odds of CV disease (OR = 0.307, *p* = 0.049), cancer (OR = 0.377, *p* = 0.025), and a DMFT score >14.99 (OR = 0.149, *p* < 0.001). Periodontal disease stage and stimulated sialometry were also significantly associated with MD adherence (*p* < 0.001 and *p* = 0.016, respectively). After adjustment, none of the systemic diseases remained statistically significant. However, for the intraoral variables, DMFT (adjusted OR = 0.486, *p* = 0.042) and periodontal disease stage (*p* < 0.001) remained significantly associated with MD adherence.

**Table 2:** Demographic characteristics and oral and systemic pathologies in the sample of 73 patients who visited the HOUB and KJCUC.

Variables	Adherence *n* (%)	Non-adherence *n* (%)	*p*	OR	BLR	OR	BLR (*p*) (intraoral examination)	OR (BLR)
Gender			0.327	0.720	0.228	0.629		
Male	25 (58.1%)	15 (50%)						
Female	18 (41.9%)	15 (50%)						
Age	60.58 ± 8.06	63.2 ± 10.79	0.239		0.444	0.922		
Comorbidities								
CV disease	5 (11.6%)	9 (30%)	0.049*	0.307	0.449	2.8		
AHT	11 (36.7%)	11 (25.6%)	0.224	0.594	0.439	0.185		
DM	13 (30.2%)	6 (20%)	0.241	1.733	0.404	0.134		
Renal disease	0 (0%)	1 (3.3%)	0.411	0.403	1	44.48		
Lung disease	2 (4.7%)	4 (13.3%)	0.185	0.317	0.999	0.000		
Cancer	0 (0%)	4 (13.3%)	0.025*	0.377	0.999	0.000		
Smoking	7 (16.3%)	5 (16.7%)	0.604	0.972	0.668	3.96		
DMFT			< 0.001*	0.149	0.249	0.852	0.042*	0.486
< 14.99	36 (83.7%)	13 (43.3%)						
> 14.99	7 (16.3%)	17 (56.7%)						
pH non-stimulated			0.948	-	0.674	0.171	0.441	0.122
< 6	4 (9.5%)	3 (10%)						
6–7	21 (50%)	16 (53.3%)						
> 7	17 (40.5%)	11 (36.7%)						
Periodontal disease stage			< 0.001*	-	< 0.001*	0.073	< 0.001*	0.268
No periodontitis	31 (72.1%)	4 (13.3%)						
Stage I	6 (14%)	4 (13.3%)						
Stage II	5 (11.6%)	11 (36.7%)						
Stage III	1 (2.3%)	11 (36.7%)						
Unstimulated sialometry			0.636	-	0.547	0.000	0.289	0.000
< 0.1mL/min	1 (2.3%)	2 (6.7%)						
0.1–0.25 mL/min	8 (18.6%)	6 (20%)						
> 0.25 mL/min	34 (79.1%)	22 (73.3%)						
Stimulated sialometry			0.016*	-	0.177	1.881	0.061	6.779
< 0.7 mL/min	0 (0%)	4 (13.3%)						
0.7–1 mL/min	8 (18.6%)	9 (30%)						
> 1 mL/min	35 (81.4%)	17 (56.7%)						
Plaque index			0.072	0.373	0.633	0.262	0.991	1.853
< 20%	15 (34.9%)	5 (16.7%)						
> 20%	28 (65.1%)	25 (83.3%)						

*P*-value (Chi-square test); OR: odds ratio; BLR: binary logistic regression; CV: cardiovascular; AHT: arterial hypertension; DM: diabetes mellitus; HOUB: Hospital of the University of Barcelona; KJCUC: King Juan Carlos University Clinic of Madrid; DMFT: Decayed, Missing, and Filled Teeth.

* Significant differences between groups.

### Intraoral examination of the HOUB population

Regarding the patients seen at the HOUB, the mean ages in the adherence and non-adherence groups were 61.36 ± 9.22 years and 64.44 ± 11.44 years, respectively. Males and females accounted for 12% and 38% in the adherence group, respectively, and 24% and 26% of the patients in the non-adherence group (Table 3).

**Table 3:** Demographic characteristics and oral and systemic pathologies in the sample of 50 patients who visited the HOUB.

Variables	Adherence *n* (%)	Non-adherence *n* (%)	*p*	OR	BLR (*p*) (intraoral examination)	OR (BLR)
Gender			0.077	0.342	0.629	
Male	6 (12%)	12 (24%)				
Female	19 (38%)	13 (26%)				
Age	61.36 ± 9.22	64.44 ± 11.44	0.292		0.444	
Comorbidities						
CV disease	2 (8%)	9 (36%)	0.017*	0.155	0.786	
AHT	4 (16%)	11 (44%)	0.031*	0.242	0.407	
DM	1 (4%)	3 (12%)	0.297	0.306	0.122	
Renal disease	0 (0%)	1 (100%)	0.312	0.490	1	
Lung disease	0 (0%)	3 (100%)	0.074	0.468	0.999	
Cancer	0 (0%)	4 (100%)	0.037*	0.457	0.999	
Smoking	2 (80%)	3 (12%)	0.637	0.638	0.668	
DMFT			0.09	0.196	0.929	0.353
< 14.99	20 (40%)	11 (22%)				
> 14.99	5 (10%)	14 (28%)				
pH non-stimulated			0.237		0.171	0.076
< 6	2 (4%)	3 (6%)				
6–7	21 (42%)	16 (32%)				
> 7	2 (4%)	6 (12%)				
pH stimulated			0.019*		0.788	0.332
< 6	3 (6%)	0 (0%)				
6–7	8 (16%)	17 (34%)				
> 7	14 (28%)	8 (16%)				
Periodontal disease stage			0.000		0.036*	0.018*
No periodontitis	18 (36%)	4 (8%)				
Stage I	4 (8%)	4 (8%)				
Stage II	2 (4%)	7 (14%)				
Stage III	1 (2%)	10 (20%)				
Unstimulated sialometry			0.186		0.998	0.998
< 0.1 mL/min	0 (0%)	3 (6%)				
0.1–0.25 mL/min	1 (2%)	2 (4%)				
> 0.25 mL/min	24 (48%)	20 (40%)				
Stimulated sialometry			0.002*		0.998	0.997
< 0.7 mL/min	0 (0%)	2 (4%)				
0.7–1 mL/min	0 (0%)	8 (16%)				
> 1 mL/min	25 (50%)	15 (30%)				
Plaque index			0.059	0.286	0.262	0.562
< 20%	10 (33.33%)	4 (13.04%)				
> 20%	15 (66.67%)	21 (65.22%)				

*P*-value (Chi-square test). OR: odds ratio; BLR: binary logistic regression; CV: cardiovascular; AHT: arterial hypertension; DM: diabetes mellitus; HOUB: Hospital of the University of Barcelona; KJCUC: King Juan Carlos University Clinic of Madrid; DMFT: Decayed, Missing, and Filled Teeth.

* Significant differences between groups.

Table 3 shows the main demographic characteristics and oral and systemic pathologies of the study population. A higher percentage of patients with systemic pathologies was observed in the non-adherence group. In the bivariate analysis, patients adhering to the MD showed lower odds of AHT (OR = 0.242, *p* = 0.031), CV disease (OR = 0.155, *p* = 0.017), and cancer (OR = 0.457, *p* = 0.037). Stimulated salivary pH and stimulated sialometry were also significantly associated with MD adherence. After adjustment, only periodontal disease stage remained statistically significant (*p* = 0.036).

### Intraoral examination of the KJCUC population

Regarding the patients seen in Madrid, the mean ages in the adherence group and non-adherence group were 59.5 ± 6.2 years and 57 ± 6.48 years, respectively. Males and females accounted for 66.7% and 33.3% in the adherence group, respectively, and 60% and 40% of the patients, respectively, in the non-adherence group (Table 4).

**Table 4:** Demographic characteristics and oral and systemic pathologies in the sample of 23 patients, those visited in the KJCUC.

Variables	Adherence *n* (%)	Non-adherence *n* (%)	*p*	OR	BLR (*p*) (intraoral examination)	OR (BLR)
Gender			0.586	1.333	0.787	
Male	12 (66.7%)	3 (33.3%)				
Female	6 (60%)	2 (40%)				
Age	59.5 ± 6.2	57 ± 6.48	0.438	-	0.578	
Comorbidities						
CV disease	3 (16.7%)	0 (0%)	0.461	0.750	0.389	
AHT	7 (38.9%)	0 (0%)	0.130	0.688	0.197	
DM	1 (5.6%)	0 (0%)	0.783	0.773	-	
Renal disease	0 (0%)	0 (0%)	-	-	-	
Lung disease	2 (11.2%	1 (20%)	0.539	0.5	0.718	
Cancer	0 (0%)	0 (0%)	-	-	-	
Smoking	5 (27.8%)	2 (40%)	0.599	0.577	0.787	
DMFT			0.048 *	0.083	0.037	0.999
< 14.99	16 (88.9%)	2 (40%)				
> 14.99	2 (11.1%)	3 (60%)				
pH non-stimulated			0.589	1.333	0.389	0.999
< 6	2 (11.8%)	0 (0%)				
6–7	0 (0%)	0 (0%)				
> 7	15 (88.2%)	5 (100%)				
Periodontal disease stage						
No periodontitis	11 (68.8%)	0 (0%)	0.009 *		<0.001*	0.997
Stage I	2 (12.5%)	0 (0%)				
Stage II	3 (18.8%)	4 (80%)				
Stage III	0 (0%)	1 (20%)				
Unstimulated sialometry						
< 0.1 mL/min	1 (5.6%)	0 (0%)	0.65		0.827	1
0.1–0.25 mL/min	7 (38.9%)	3 (60%)				
> 0.25 mL/min	10 (55.6%)	2 (40%)				
Stimulated sialometry						
< 0.7 mL/min	0 (0%)	2 (40%)	0.019 *		0.119	1
0.7–1 mL/min	8 (44.4%)	1 (20%)				
> 1 mL/min	10 (55.6%)	2 (40%)				
Plaque index						
< 20%	5 (27.8%)	1 (20%)	0.608	0.65	0.766	1
> 20%	13 (72.2%)	4 (80%)				

*P*-value (Chi square test); OR: Odds Ratio; BLR: Binary logistic regression;

* Significant differences between groups.

Table 4 shows the main demographic characteristics and oral and systemic pathologies of the 23 patients. A higher percentage of patients with systemic pathologies was observed in the adherence group.

Regarding the intraoral data collected, better results in terms of oral health were observed in the adherence group. In the bivariate analysis, DMFT (*p* = 0.048), periodontal disease stage (*p* = 0.009), and stimulated sialometry (*p* = 0.019) were statistically significant. In the adjusted BLR, which included systemic diseases and intraoral examination, only the periodontal disease stage remained statistically significant (*p* < 0.001).

## Discussion

This study aimed to investigate whether greater adherence to the MD, that is, an antioxidant and anti-inflammatory diet, is associated with a lower risk of developing oral or systemic pathologies.

Using the MEDAS questionnaire, medical history, and intraoral examination, we found that systemic pathologies such as cancer and CV disease had a significant negative relationship with MD adherence. Additionally, stimulated pH, periodontal disease stage, and stimulated sialometry were also correlated with MD adherence. Although the other pathologies were not significant, they presented a greater risk of occurrence when there was no adherence to the MD.

Regarding systemic pathologies, our study found a significant association between higher adherence to the MD and a lower prevalence of cancer. This finding is consistent with previous evidence indicating that diets rich in fruits, vegetables, and whole grains, and low in red and processed meat, are associated with a reduced risk of several common cancers, including lung, esophageal, stomach, and colorectal cancers [8, 9, 23]. Supplementation with these nutrients does not appear to provide the same preventive effects [8, 9, 23]. It is recommended to consume foods based on whole grains and limit the consumption of red and processed meat. Alternatively, legumes which are rich in nutrients, serve as a healthier source of protein and may help reduce the risk of cancer [23]. Diets high in fat are associated with increased cancer risk, but some fats, such as omega-3 fatty acids in fish and MUFA or PUFA (polyunsaturated fatty acid) in olive oil, tend to reduce the risk [9, 20, 24].

Moreover, the role of diet is crucial in the management of type II DM, not only for prevention. Treatment should be individualized considering the dietary patterns, goals, and lifestyles of each patient [25]. The MD, compared with a high-protein, low-carbohydrate diet, may lead to a greater decrease in glycosylated hemoglobin (HbA1c), up to 0.30–0.47%. Although our study did not find significant results, there was a higher percentage of patients with DM in the non-adherence group. The protective potential of the MD against diabetes can be attributed to its anti-inflammatory and antioxidant properties, primarily due to the presence of protective nutrients such as fiber, vitamins, and minerals. Additionally, it is characterized by a lower intake of pro-inflammatory foods, including saturated and trans fatty acids, refined sugars, and starches [26]. MUFAs and PUFAs from plants, in the form of olive oil, nuts, and seeds, can improve glucose metabolism and insulin sensitivity. Furthermore, their consumption instead of saturated or trans fatty acids can reduce the risk of type II DM by up to 83% over an average of 4.4 years, even without caloric restriction [25].

The healthy dietary pattern is characterized by a high consumption of fruits, vegetables, nuts, legumes, and whole grains, such as the MD. Moreover, a reduced intake of salt, sugar-sweetened beverages, and meat has been associated with a lower incidence of CKD and albuminuria. A cohort study conducted in 2403 patients with a glomerular filtration rate between 20 and 70 mL/min concluded that increased adherence to a healthy diet with characteristics similar to the MD is associated with a lower risk of CKD progression and death among patients with CKD. Currently, the consumption of vegetables and whole-grain products is recommended, as they are not associated with increased serum potassium levels. This is because the bioavailability of potassium and phosphorus in plant foods is less than 50%, whereas in ultra-processed foods it is 100%. The high vegetable content present in the MD is associated with slower progression of CKD, a reduction in constipation, and an improvement in the intestinal microbiota due to the higher amount of fiber consumed [27]. Although there were no significant findings in our study, there was a higher percentage of patients with CKD in the non-adherence group.

On the other hand, some of the components of the MD reduce blood pressure, improve the lipid profile, and are associated with reduced markers of vascular inflammation. Our results show a lower percentage of AHT with MD adherence, in agreement with a great deal of evidence. In the study by Estruch et al. [28], participants at high CV risk who adhered to the MD by consuming more virgin olive oil and nuts had a decrease in blood pressure. In the MD, regular but moderate intake of wine during meals (1–2 glasses/day) is recommended. The benefits of wine consumption, especially red wine, are due to polyphenol compounds found in plants such as red grapes and their derivatives. These exhibit potent anti-inflammatory and antioxidant properties due to the antioxidant enzymes present in polyphenols [29]. The “Prevención con Dieta Mediterránea” (PREDIMED) trial has demonstrated beneficial effects of an MD enriched with extra virgin olive oil in the primary prevention of CV diseases such as atherosclerosis. It seems to be related to an improved lipid profile and lower atherogenic risk [28]. An observational cohort study by Sofi et al. [30] and a secondary prevention trial by De Lorgeril et al. [31] have concluded that increasing adherence to the MD is consistently beneficial with respect to CV risk. In the study by Brunner et al. [32], supporting our significant results on CV disease, rates of nonfatal myocardial infarction and coronary death were lower among those with a healthy dietary pattern, distinguished by high consumption of fruits and vegetables, polyunsaturated oils, high-fiber cereals, and low consumption of red meat, saturated fats, and refined carbohydrate foods.

Fruits and vegetables, present in MD, may provide fiber and antioxidants, which may reduce oxidative stress-related inflammatory disease. Vitamin C, β-carotene, magnesium, and selenium are associated with reduction in asthma prevalence and may prevent or limit an inflammatory response in the airways by reducing reactive oxygen species and inhibiting lipid peroxidation [33]. Although in our study there were no significant findings, there was a higher percentage of patients with asthma in the non-adherence group. In the study by Barros et al. [34], daily fruit intake above 300 g decreased the risk of uncontrolled asthma by 71%. Intake of MUFAs has been associated with an increased risk of allergic sensitization, while saturated fatty acids such as those found in dairy products have been associated with a lower risk of asthma [35].

Regarding intraoral pathologies, numerous studies have shown that sugars are the most important factor in the development of caries [2]. Although our study did not show significant findings on caries, DMFT was significant in the Madrid group of patients, and there was a higher percentage of patients with caries in the non-adherence group. Both the frequency and total consumption are associated with caries levels. It has been observed that if sugar is consumed during meals and no more than four times a day, it does not increase the risk of caries. Therefore, the progression of caries stops when sugar is removed from the diet [2]. Acidogenic foods, such as carbohydrates and sugars, acidify the salivary pH, causing changes in the oral microbiota that facilitate the adhesion of bacteria to the tooth surface, initiating the process of cariogenesis. Starch is considered to be of low cariogenicity; when cooked, it is between one-third and one-half as cariogenic as sucrose. However, mixtures of starch and sucrose are potentially more cariogenic than sugars alone [2]. These findings are consistent with previous reports showing a significant difference in stimulated pH between the groups visited in Barcelona, and a significant difference in stimulated sialometry in both the Barcelona and Madrid patients. Marqués-Martínez et al. [35] found an association between adherence to the MD and the prevalence, extent, and severity of dental caries. Promoting healthy dietary patterns and emphasizing foods that can protect against caries is crucial. Additionally, the use of biomimetic hydroxyapatite-based toothpastes represents a novel approach to managing the disease [36].

Nutrition has been associated with periodontal disease. Periodontal disease is considered an inflammatory disease, and some nutrients are involved in the modulation of inflammation, such as vitamin C, vitamin E, and carotenoids. In this study, periodontal disease showed a significant relationship with MD adherence. Healthy dietary practices have been associated with reduced plaque formation, calculus, and periodontal disease. A poor-quality diet is associated with significant calculus formation (OR = 1.54). A prospective study revealed that those who consume large amounts of whole grains are 23% less likely to have periodontitis. It has also been analyzed whether milk and dairy products are related to the risk of periodontitis, showing a 20% lower prevalence in higher consumers compared to those in the lowest consumption quintile [19]. Olive oil, essential in the MD, has been significantly associated with a lower prevalence of periodontitis. It plays an important role in host immune resistance to infection [37]. In terms of treatment, the efficacy of scaling and root planing is well established; however, its main limitation is bacterial recolonization following the procedure. To address this issue, alternative therapies have been introduced. Probiotic therapy is gaining popularity due to its ability to avoid the side effects associated with antibiotics. Probiotics can compete with pathogens for nutrients and adhesion to epithelial cells, produce antimicrobial substances, modulate the immune response, and enhance mucosal barrier function [38]. Recently, postbiotics have been introduced as a novel technique. With their antioxidant properties and food origin, postbiotics accelerate healing by reducing the bacterial load in implant tissues [39, 40].

This study had several limitations. First, the small sample size: 232 patients were recruited, of whom only 73 had their oral cavity examined, with only 23 recruited in Madrid. Second, the small difference in sample size between groups: for systemic diseases, the group with adherence to the MD consisted of 124 patients, while the group without adherence to the MD consisted of 108 patients. In the oral cavity study, the groups were reduced to 43 and 30 patients, respectively. Finally, self-reporting bias should be considered, as patients may have overestimated the quality of their diet when completing the questionnaire in the presence of a healthcare professional. However, this study supports the published evidence. A larger sample size would be necessary to continue the study and obtain more significant results.

In conclusion, higher adherence to the MD, characterized by its antioxidant and anti-inflammatory properties, was associated with a lower prevalence of several oral and systemic diseases. Significant associations were observed with CV disease, cancer, periodontal disease stage, stimulated salivary flow, and salivary pH. These findings support a potential role of the MD in promoting oral and systemic health; however, owing to the cross-sectional design of the present study, causal relationships cannot be established.

## Data Availability

All relevant data are provided within the article.
